# Lumbar Spondylolysis: Future Perspectives

**DOI:** 10.31662/jmaj.2024-0202

**Published:** 2024-09-27

**Authors:** Yuki Taniguchi, Yasushi Oshima, Sakae Tanaka

**Affiliations:** 1 Surgical Center, The University of Tokyo Hospital, Tokyo, Japan; 2 Department of Orthopedic Surgery, The University of Tokyo Hospital, Tokyo, Japan

**Keywords:** isthmic spondylolisthesis, spondylolysis, pars repair, pars interarticularis

Spondylolysis is a condition characterized by a defect or fracture of the pars interarticularis and is one of the most common causes of low back pain in the pediatric and adolescent population. It most commonly occurs at L5 (85%-95%), particularly impacting individuals engaged in athletic activities. While its cause remains mostly unknown, spondylolysis likely occurs because of a combination of mechanical stress and genetic predisposition. Since lumbar spondylolysis can progress to isthmic spondylolisthesis in the adulthood, leading to chronic low back pain and/or radiculopathy, it is crucial to administer appropriate treatment during childhood and adolescence. Additionally, due to the high prevalence of lumbar spondylolysis, extensive research has been conducted to date on its epidemiology, natural history, and treatment options. However, many unresolved issues remain, which should be addressed to improve the prognosis of young patients.

Several imaging modalities are utilized in the management of spondylolysis, which include radiographs, computed tomography (CT), magnetic resonance imaging and bone scintigraphy. Among them, CT offers the advantage of high-resolution imaging of bony anatomy and the ability to accurately stage spondylolysis and assess healing in chronic defects. Since CT has been identified as the best modality for tracking healing following initial diagnosis, it is not uncommon to perform multiple CT scans on the same patient. However, CT inherently has the disadvantage of radiation exposure. Particularly, due to the fact that lifetime cancer mortality risks attributable to radiation from a pediatric CT examination are estimated to be much higher than adults, CT scans in childhood and adolescence should be avoided as much as possible ^(1)^. This is a major challenge in the current clinical practice for musculoskeletal diseases, particularly emphasizing the need for the development of new imaging modalities that do not require radiation exposure but excel in depicting bone structures.

In the treatment of lumbar spondylolysis, achieving bone union during childhood and adolescence is considered desirable to prevent progression to isthmic spondylolisthesis in the future. However, despite the presence of pseudoarthrosis in the pars defect, surgical treatment is currently limited to patients with persistent low back pain or restrictions in sports activities, and conservative therapy is the mainstay of treatment. This is because the discomfort usually subsides, allowing patients to resume athletic activities, even in the presence of pseudoarthrosis at the site of the pars defect. Additionally, the need to restrict sports activities for a certain period after surgery further supports the emphasis on conservative therapy. However, recent studies have shown that the progression to isthmic spondylolisthesis in the natural course of lumbar spondylolysis is more frequent than previously thought. Aoki et al. demonstrated an age-dependent increase in progression to spondylolisthesis, with as high as 90% of bilateral spondylolysis patients older than 60 years old exhibiting spondylolisthesis ^(2)^. Considering the high rate of progression to isthmic spondylolisthesis in adulthood in the natural course, there may be a case for considering surgical intervention during childhood or adolescence even in asymptomatic patients with lumbar spondylolysis. In this issue of JMA journal, Himi et al.^(3)^ highlighted the elevated nonunion rate of pars fractures in patients younger than 10 years old. Given the strong bone healing capacity in this age group, prophylactic pars repair may be particularly advisable for these pediatric patients more proactively than it is currently practiced.

Surgery is indicated for patients who do not have symptom improvement with conservative treatment and continue to have pain interfering with daily activity or ability to return to sports. A total of 5% of athletes are reported to fail conservative treatment and require surgery. Posterolateral fusion was once the only method of choice; however, direct pars repair is the mainstay of treatment and the first-line option today, because spinal fusion for spondylolysis reduces the number of mobile segments in the lumbar spine and has the potential to induce adjacent segment disease. To date, a variety of methods for direct pars repair have been developed. Mohammed et al. conducted meta-analysis comparing the four most common direct repair techniques, which included Buck repair (direct pars lag screw), Scott repair (segmental wire fixation), Morscher repair (screw hook), and pedicle screw-based techniques, and concluded that pedicle screw techniques had the highest fusion rate and lowest complication rate, with the Buck repair providing the second-best results ^(4)^. Recently, reports on the minimally invasive pars repair technique have emerged ^(5)^. Minimally invasive surgery for lumbar spondylolysis is particularly promising for athletes as it minimizes damage to soft tissues, such as paraspinal muscles. Additionally, pars repair surgery may be especially compatible with navigation-assisted or robot-assisted minimally invasive surgery, given the potential challenges of direct screw placement techniques like the Buck method owing to the difficulty of screw insertion. However, in minimally invasive pars repair technique, there is a possibility that thorough debridement of the fibrous or cartilaginous tissue and the sclerotic bone margins at the pars defect and sufficient bone grafting, which are considered essential for achieving firm bone fusion, can be insufficient due to the limited surgical field. Therefore, the long-term outcomes and reports from multiple institutions are awaited to fully evaluate the efficacy of minimally invasive pars repair techniques. The establishment of the efficacy of minimally invasive techniques may contribute to the aforementioned expansion of the surgical indications.

In summary, we have addressed the unresolved challenges in the management of lumbar spondylolysis in young patients with the goal of refining our daily clinical practice and subsequently improving patient outcomes by systematically addressing these issues in the near future.

## Article Information

### Conflicts of Interest

None

### Author Contributions

YT wrote the original draft. YO and ST reviewed the draft critically for important intellectual content. All authors finally approved version to be published.

### Approval by Institutional Review Board (IRB)

Not applicable.

### Disclaimer

Sakae Tanaka is one of the Editors of JMA Journal and on the journal’s Editorial Staff. He was not involved in the editorial evaluation or decision to accept this article for publication at all.
